# Reanalysis of Association of Pro50Leu Substitution in Guanylate Cyclase Activating Protein-1 With Dominant Retinal Dystrophy

**DOI:** 10.1001/jamaophthalmol.2019.4959

**Published:** 2019-12-05

**Authors:** Omar A. Mahroo, Gavin Arno, Rola Ba-Abbad, Susan M. Downes, Alan Bird, Andrew R. Webster

**Affiliations:** 1 Genetics Service, Moorfields Eye Hospital, London, United Kingdom; 2 Institute of Ophthalmology, University College London, United Kingdom; 3 Section of Ophthalmology, King’s College London, St Thomas’ Hospital Campus, London, United Kingdom; 4 Department of Physiology, Development and Neuroscience, University of Cambridge, United Kingdom; 5 Oxford Eye Hospital, Oxford University Hospitals National Health Service Foundation Trust, Oxford, United Kingdom; 6 Nuffield Laboratory of Ophthalmology, Department of Clinical Neurosciences, University of Oxford, Oxford, United Kingdom

## Abstract

**Importance:**

As genetic and genomic screening is becoming more widely accessed, correctly distinguishing pathogenic from nonpathogenic variants is of increasing relevance.

**Objective:**

To reevaluate a previously reported family in whom the p.(Pro50Leu) variant in the gene *GUCA1A* was associated with a dominant retinal dystrophy, in light of new examination findings in the proband’s daughter.

**Design, Setting, and Participants:**

A genetic study relating to a family with an inherited retinal dystrophy was performed at the retinal genetics service of Moorfields Eye Hospital from October 27, 2009, to May 23, 2019, after the proband’s daughter underwent fundus examination.

**Main Outcomes and Measures:**

Results of sequencing of X chromosome–linked retinitis pigmentosa genes in the proband and specific analysis of the repetitive ORF15 region of the *RPGR* gene.

**Results:**

A frame-shifting single-nucleotide deletion was found in the ORF15 exon of *RPGR* (GRCh37 [hg19] x:38145160delT; NM_001034853.1: c.3092delA p.[Glu1031Glyfs*58]), which may be associated with the loss of 121 amino acid residues at the carboxyl terminus of the protein. The p.(Pro50Leu) variant in *GUCA1A* was also found to be too common in a publicly available genome database to be a fully penetrant cause of a dominant retinal dystrophy.

**Conclusions and Relevance:**

The phenotype in the family is now associated with the variant in *RPGR*. The findings suggest that the p.(Pro50Leu) variant in *GUCA1A* should not be regarded as pathogenic. This report also highlights the relevance of examining relatives, of reevaluating diagnoses in light of new data, and of considering X chromosome–linked inheritance in apparently autosomal dominant pedigrees unless there is clear male-to-male transmission.

## Key Points

**Question:**

Is there evidence that the Pro50Leu substitution in guanylate cyclase activating protein-1 (encoded by the gene *GUCA1A*) is associated with a dominant retinal dystrophy?

**Findings:**

In this cross-sectional study reevaluating the original published study of a family after examination of another family member and further genetic testing, a pathogenic variant in the X chromosome–linked *RPGR* gene was found. Also, publicly available genomic data show that the variant in *GUCA1A* is too common to cause a dominant retinal dystrophy.

**Meaning:**

The p.(Pro50Leu) variant in *GUCA1A* should not be considered a pathogenic variant.

## Teaser

This cross-sectional study reevaluates a previously reported family in whom the p.(Pro50Leu) variant in the gene *GUCA1A* was associated with a dominant retinal dystrophy, in light of new examination findings in the proband’s daughter.

## Introduction

The gene *GUCA1A* (OMIM *600364) encodes the protein guanylate cyclase activating protein-1, a calcium-sensitive protein integral to maintaining cyclic guanosine monophosphate levels in the outer segment of photoreceptors. Variants in this gene may be associated with a dominantly inherited cone dystrophy, largely by affecting calcium sensitivity.^1,2,3,4^ In 2001, 3 families with variants in *GUCA1A* were reported in this journal.^2^ Two families had the p.(Tyr99Cys) variant, with a relatively consistent phenotype. The third family had the p.(Pro50Leu) variant and exhibited a more variable phenotype (the proband had a cone dystrophy, but an affected relative had features consistent with retinitis pigmentosa). Since that publication, this family has undergone more detailed investigation, the results of which are described in the present report; we now associate their phenotype with a disease-causing variant in *RPGR* (OMIM *312610, coding for the retinitis pigmentosa [RP] GTPase regulator protein, thought to facilitate protein trafficking in photoreceptors) and not *GUCA1A*. Two of us (S.M.D. and A.B.) were also contributing authors of the original report.^2^

## Methods

### Clinical Examination and Imaging

This study was performed from October 27, 2009, to May 23, 2019, after the original evaluation of the family, because the proband’s daughter underwent a fundus examination (see Results). A fundal examination was performed at the slitlamp after mydriasis, and color fundus images and red-free images were taken from both eyes. The study was approved by Moorfields Eye Hospital and Northwest London Research Ethics Committee, and conformed to the tenets of the Declaration of Helsinki.^5^ Participants provided written informed consent.

### Genetic Testing and Estimating Population Prevalence of the *GUCA1A* Variant

Sanger sequencing of DNA from the proband was performed to screen for the *RPGR* and *RP2* (OMIM *300757) genes (Manchester Genomic Diagnostics Laboratory). The publicly available gnomAD data set (https://gnomad.broadinstitute.org/; accessed May 23, 2019) was used to estimate the prevalence of the p.(Pro50Leu) variant in *GUCA1A*.

## Results

**Figure 1.**
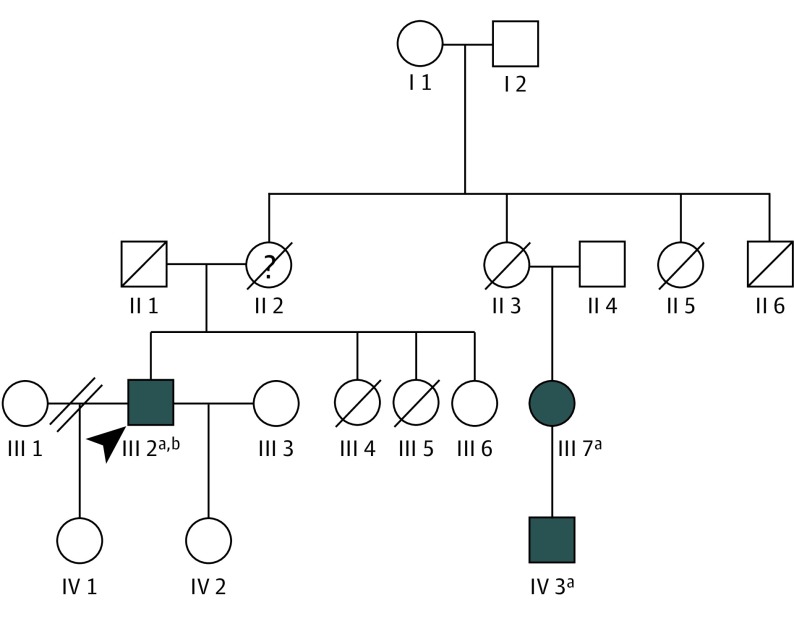
Pedigree of Family Presence of affected individuals in more than 1 generation is suggestive of autosomal dominant inheritance, as previously assumed. However, because there is no male-to-male transmission, X chromosome–linked inheritance is not excluded. The proband (denoted by the arrowhead) had a cone dystrophy, whereas individual IV 3 had a retinitis pigmentosa phenotype (this individual was labeled III 10 in the previous publication; a generation was missed owing to incomplete family information). ^a^Individuals found in the previous report to have the p.(Pro50Leu) variant in *GUCA1A*. One of the daughters of the proband (IV 2) was subsequently found to have a tapetal reflex (Figure 2), raising the possibility of an X chromosome–linked retinopathy. ^b^The presence of the *RPGR* variant was found in the proband in the present study. Other family members were no longer able to be contacted to check segregation of this allele.

**Figure 2.**
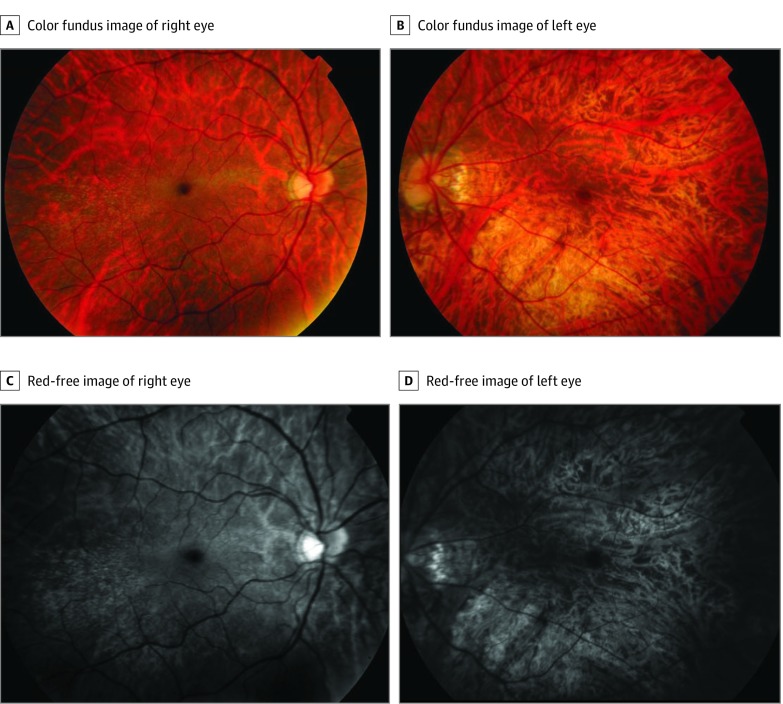
Fundus Imaging From the Daughter of Proband in Figure 1, Aged 26 Years A, Color fundus image from the right eye showing a tapetal reflex. B, Color fundus image from the left eye showing atrophic changes consistent with pathologic myopia. C, Red-free image of the right eye in which the tapetal reflex is more evident. D, Red-free image of the left eye. The patient’s corrected visual acuity was 20/30 OD and 20/1800 with amblyopia OS; refraction was −9.75 diopters in the right eye and −16.50 diopters in the left eye.

Figure 1 depicts the pedigree (updated from the previous publication^2^). The proband exhibited a cone dystrophy, whereas his relative (IV 3) had features consistent with RP. One of the proband’s daughters was examined subsequently in our service and found to have high myopia and a tapetal reflex (Figure 2 shows color fundus images and red-free images). The latter sign, a known carrier phenotype in X chromosome–linked RP, raised the likelihood of an X chromosome–linked retinal dystrophy.

Results of initial screening of *RPGR* and *RP2* (2 X chromosome–linked genes in which variants can give rise to RP) using Sanger sequencing were negative. A subsequent reanalysis of the original sequencing data of the ORF15 exon of *RPGR* demonstrated a frame-shifting single-nucleotide deletion (GRCh37 [hg19] x:38145160delT; NM_001034853.1: c.3092delA p.(Glu1031Glyfs*58)), which may be associated with the loss of 121 amino acid residues at the carboxyl terminus of the protein. This variant has been previously reported in association with X chromosome–linked retinal disease.^6^ Interrogation of the gnomAD data set revealed an allele frequency for the p.(Pro50Leu) variant in *GUCA1A* of 0.12% (337 of 282 870 alleles), reaching a frequency in Northwest European alleles of 0.22% (114 of 50 812 alleles).

## Discussion

Based on our findings, we now believe that the phenotype in this family is due to the pathogenic variant in *RPGR* and not the p.(Pro50Leu) variant in *GUCA1A*. The latter is too common to be a pathogenic, fully penetrant, dominantly acting variant. In addition, reported pathogenic changes in the gene are clustered in regions affecting calcium sensitivity^3^; this substitution occurs in an earlier part of the protein, and biochemical studies have shown that the p.(Pro50Leu) variant is not associated with calcium sensitivity (although the protein shows less thermal stability and greater susceptibility to protease digestion).^7^ Pathogenic variants in *GUCA1A* also are not associated with the RP phenotype, which was exhibited by an affected relative of the proband. Taken together with our updated findings of a variant in *RPGR* in the reported family, we conclude that there is little evidence for the pathogenicity of the *GUCA1A* variant.

*RPGR* is the gene most frequently associated with X chromosome–linked retinal dystrophy. Male patients with pathogenic variants in this gene usually have RP (electrophysiologically a rod-cone dystrophy), but affected individuals can display a cone or cone-rod dystrophy.^8^ Female carriers can show a tapetal reflex or disease phenotypes of varying severity, including a phenotype as severe as that seen in affected males.^9^ Screening of the ORF15 exon, where many pathogenic variants lie,^6^ can be challenging owing to a repetitive nucleotide sequence; hence, variants can be missed, even in the context of whole-genome sequencing. Variants in this exon also are more often associated with a cone or cone-rod dystrophy phenotype (compared with changes in other parts of the gene) and have been shown occasionally to be associated with both cone dystrophy and RP phenotypes in the same family,^8^ as in this pedigree. Discordant phenotypes have been reported in fraternal twins with the same pathogenic variant as that found in the family we studied.^10^

As genetic and genomic testing become more common, distinguishing nonpathogenic and pathogenic variants is increasingly relevant. One key piece of evidence is a report of the variant cosegregating with the disorder in question.^11^ Such reports occasionally are false positives, as is the case here, and its correction is therefore necessary.

### Limitations

A limitation of the study was that we were unable to fully check segregation of the *RPGR* variant in the family, as other family members were no longer able to be contacted. However, as noted above, the variant has been associated with both cone-rod dystrophy and RP phenotypes, and so would be consistent with the phenotype previously reported in affected family members.

## Conclusions

Our report also highlights a number of points of wider relevance. Investigators should be wary of prior reports of pathogenic variants in publicly available databases if they are based on 1 family, especially when testing was performed with earlier methods. Second, it is advisable, when feasible, for genetic investigators to reevaluate their published reports on families and prior diagnoses in light of new findings, such as a high prevalence in the gnomAD data set, or new examination findings of family members. Third, families with X chromosome–linked disease can be misinterpreted as having autosomal dominant inheritance owing to female family members being affected; X chromosome–linked inheritance should be considered in all families for which multiple generations of family members are affected unless there is clear male-to-male transmission. Correctly identifying *RPGR*-related disease likely is of increasing relevance because this is now the subject of gene replacement trials.^12^
